# Photocatalytic Oxidation Reactions Mediated by Covalent Organic Frameworks and Related Extended Organic Materials

**DOI:** 10.3389/fchem.2021.708312

**Published:** 2021-06-24

**Authors:** José Alemán, Rubén Mas-Ballesté

**Affiliations:** ^1^ Department of Organic Chemistry (Module 1), Facultad de Ciencias, Universidad Autónoma de Madrid, Madrid, Spain; ^2^ Institute for Advanced Research in Chemical Sciences (IAdChem), Universidad Autónoma de Madrid, Madrid, Spain; ^3^ Department of Inorganic Chemistry (Module 7), Facultad de Ciencias, Universidad Autónoma de Madrid, Madrid, Spain

**Keywords:** covalent organic frameworks, photocatalysis, synthesis, oxidation, oxygen evolution reaction

## Abstract

Covalent Organic Frameworks (COFs) and related extended organic materials have been widely used as photocatalysts in the last few years. Such interest arises from the wide range of covalent linkages employed in their construction, which offer many possibilities to design extended frameworks and to link photoactive building blocks. Thus, the potential utility of predesigned organic photoactive fragments can be synergistically added to the inherent advantages of heterogeneous catalysis, such as recyclability and easy separation of catalyst. In this overview, the current state of the art on the design of organic materials for photocatalytic oxidation reactions will be presented. The designing process of these materials is usually conditioned by the generally accepted concept that crystallinity and porosity defines the quality of the heterogeneous catalysts obtained. The care for the structural integrity of materials obtained is understandable because many properties and applications are intimately related to these features. However, the catalytic activity does not always directly depends on these characteristics. A critical compilation of the available literature is performed in order to offer a general perspective of the use of COFs and Covalent Triazine Frameworks (CTFs) in photocatalytic oxidation processes, including water oxidation, which constitute an important outcome relevant to artificial photosynthesis.

## Introduction

Covalent Organic Frameworks (COFs) is an emerging family of laminar or 3D porous materials only composed by organic linkers connected through covalent bonds. Thus, they are constituted by earth abundant elements, and show high stability, low toxicity, low density and adjustable pore size, chemical structure and functionality. Therefore, applications of COFs have been reported in a variety of areas, such as storage and separation of gases, energy storage, drug delivery, proton conduction, optoelectronics and catalysis (Ding and Wang, 2013). Another family of materials closely related are Covalent triazine-based frameworks (CTFs) which are assembled through nitrile trimerization. CTFs have been identified for photocatalytic applications (López-Magano et al., 2020a). Conjugated organic frameworks offer the opportunity to design catalytic heterogeneous systems allowing easy separation of the catalyst as well as their recyclability. Furthermore, the photocatalytic performance of COFs, and their amorphous analogs, has been induced by designing highly conjugated extended structures or by incorporating pre-dedetermined photoactive units into the framework. Therefore, photocatalytic applications of reticular organic materials have been reported in the areas of organic chemical synthesis, degradation of pollutants, CO_2_ reduction and water splitting, among others (Bonesi et al., 2006; Dad’ová et al., 2012; Guo et al., 2014; Fang et al., 2015; Ma et al., 2016; Shaw et al., 2016; Mandal et al., 2017; Zhu and Zhang, 2017; Samanta et al., 2019; López-Magano et al., 2020a; Song et al., 2020; Wang et al., 2020).

This overview presents a brief description of the state of the art on photocatalytic oxidation reactions using porous organic materials. In particular, two different approaches with distinct requirements are shown. First, oxygen molecule activation is a valuable synthetic tool to have access to products of diverse complexity through an environmentally benign process that uses oxygen as final oxidant and light as energy source to overcome thermodynamic and/or kinetic barriers (Shaw et al., 2016). On the other hand, oxygen evolving water oxidation is a more complex process, which implies the transfer of four electrons coupled to the formation of a O-O bond. In fact, this transformation is the most challenging half reaction of water splitting due to the thermodynamic and kinetic challenges that presents (Song et al., 2020). Very rencently, as shown in the next paragraphs, organic materials started to be considered as good platforms to perform this process in a photocatalytic manner. Overall, the reports compiled in this short overview represents the initial steps of a promising research field with deep social and economic implications.

## Aerobic Photo-Oxidation of Organic Molecules

**FIGURE 1:**
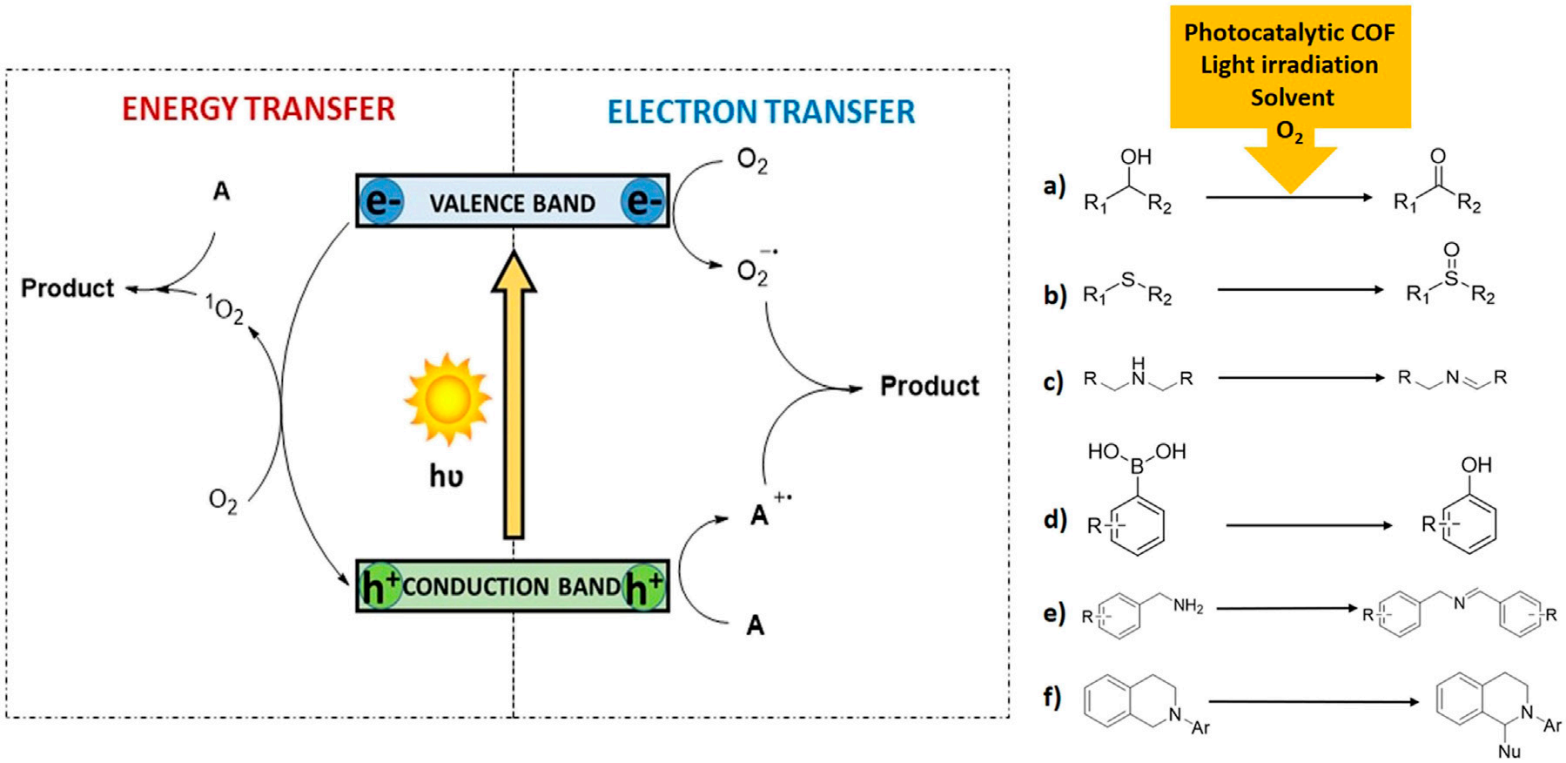
Two different mechanism for oxidation reaction using organic materials **(left)** and main organic erobic oxidations of organic substrates **(right)**.

In industry, oxidation reactions are employed for the obtention of aldehydes, epoxides, alcohols, and ketones, among others. The use of organic peroxides, permanganates and dichromates have traditionally been employed in these oxidations, and therefore harmful byproducts are produced. On the other hand, the use of molecular oxygen as final oxidant and heterogeneous photocatalysts results in more sustainable and greener reactions (Guo et al., 2014). The use of oxygen to perform this transformation is usually described by two plausible mechanisms: an electron transfer or an energy transfer (Figure 1, left). In the former, the photocatalyst undergoes a charge separation under light irradiation. Then, substrate **A** is oxidized to its corresponding cationic radical **A** ^**·+**^ by the formed holes of the photocatalyst, while O_2_ is reduced to O_2_^−·^ by the photogenerated electrons (electron transfer mechanism). Finally, the **Product** is formed when the cationic radical **A** ^**·+**^ intermediate, reacts with the active oxygen species. In the later, conversion of ^3^O_2_ into ^1^O_2_ takes place as a consequence of an energy transfer process between the triplet excited state of the catalytic fragment and oxygen in its ground state. Then, singlet oxygen reacts with substrate **A** and generates the final **Product**. The differentiation of one or another pathway can be precluded using selective scavengers [e.g., 1,4-diazabicyclo[2.2.2]octane (DABCO) for singlet oxygen (Bonesi et al., 2006) or 1,4-dimethoxybenzene for superoxide radical anion (Dad’ová et al., 2012)]. Interestingly, is common to observe that both mechanisms can operate concurrently. In the following lines, we will mention the main aerobic oxidation transformations using photocatalytic COFs (Figure 1, right).

a) **Photocatalytic oxidation of alcohols:** The first example to perform this reaction was reported in 2017 for a thiophene-containing Covalent Triazine Framework (CTF) (**CTF-Th@SBA-15**) (Huang et al., 2017). Later, a hybrid material consisting TiO_2_ nanobelts coated with an imine-based COF (**TiO**_**2**_ **@COF-3**) was used to perform benzylic alcohol oxidation under visible light irradiation (Lu et al., 2019). This material presented an enhanced photocatalytic activity related to the possibility of charge transfer from COF to TiO_2_. On the other hand, in 2020, a hybrid MOF–COF, **NH**_**2**_ **-MIL-125@TAPB-PDA-3**, shown similar photocatalytic properties (Lu et al., 2020).
b) **Selective Sulfoxidation Reactions:** Sulfoxides are prevalent structures in agriculture and pharmaceutical industries (Wojaczyńska and Wojaczyński, 2010). Therefore, a common model reaction studied in photocatalytic systems is the selective sulfoxidation of organic sulfides. Three different undecorated imine-based materials were published by our group (a 3D-COF, a layered-COF, and a spherical-COF), which showed good results in the photocatalytic sulfoxidation under green reaction conditions (water and ethanol as solvents) (Jiménez-Almarza et al., 2019). In addition, 2 *N*, *N*′-bicarbazole-based CTFs, **BC-CTF**, and **Ph-BC-CTF,** were able to perform the same reaction (Yan et al., 2019). Later on, a 2D and a 3D Pd-containing porphyrinic COFs, **2D-PdPor-COF** and **3D-PdPor-CO**F, performed in a very efficient manner the oxidation of sulfides (Meng et al., 2020). Furthermore, **h-LZU1**, a nanostructured COF, showed a slightly worse recyclability and selectivity in photocatalytic sulfoxidation of organic sulfides (Liu et al., 2020a). Very recently, our group reported the attachment of Pt^II^-hydroxyquinoline fragments in defective sites of an imine-based COF structure. This metal fragment acted as photocatalytic center showing TON up to 8,000 with excellent stability and recyclability (López-Magano et al., 2020b).
c) **Dehydrogenation of Secondary Amines:** The selective oxidative dehydrogenation of secondary amines to imines is feasible due to the impossibility of the substrates to generate nitriles, but is limited by the steric hindrance around the N−H bond. Furthermore, the presence of two different α-CH bonds in asymmetric dibenzylamines can compromise the chemoselectivity, since it can be found two different oxidized products. As a consequence, the oxidation of symmetric dibenzylamine is the chosen reaction for the study of these coupling reactions, while asymmetric dibenzylamine examples still remain challenging (Chen et al., 2019a; Chen et al., 2019b). Both oxygen anion radical and singlet oxygen have been found as reactive oxygen species in this process. In addition, **Por-sp** ^**2**^ **c-COF** (cyanovinylene-based porphyrin-containing COF) was used for the synthesis of imine under photocatalytic conditions (Yan et al., 2019; Meng et al., 2020). Moreover, **Por-sp** ^**2**^ **c-COF** was also employed for the oxidation of secondary amines, using TEMPO as co-Catalyst, allowing the use of red light in a two-photon absorption process (Shi et al., 2020).
d) **Oxidation of Arylboronic Acids:** Generation of superoxide radical anion results in the oxidation of arylboronic acids to phenols (Zou et al., 2012). This reaction has been achieved by the following materials: a) three new benzoxazole-linked COFs, (**LZU-190**) obtained in 2018 (Wei et al., 2018), b) an imine-linked COF obtained in 2019 through the self-condensation of an aldehyde and amine containing tetratopic building block, (Wojaczyńska and Wojaczyński, 2010; Lu et al., 2019; Lu et al., 2020), c) three imine-based undecorated COFs with different architectures and morphologies (Huang et al., 2017).
e) **Oxidative Coupling:** Firstly, the coupling imine product from primary amines was obtained using **Por-sp** ^**2**^ **c-COF** (porphyrin-containing COF) as photocatalyst combined with TEMPO as co-catalyst and oxygen as the final oxidant (Chen et al., 2019a; Chen et al., 2019b; Liu et al., 2020a; López-Magano et al., 2020b). Also, benzylamine was oxidized using **TFPT-BMTH** (hydrazone-based COF) and water as solvent. It should be highlighted that the use of more stable materials is needed for this reaction, since amines are employed as reagents, (Liu et al., 2020b), which due to their nucleophilicity can act against the structural integrity of the material (Luis-Barrerra et al., 2019).
f) **Cross-Dehydrogenative Coupling (CDC):** In 2016, a CDC reaction between different nucleophiles (e.g., nitro-derivatives and ketones) and *N*-aryl-tetrahydroisoquinolines (THIQs) was achieved under **TFB-COF** (an acylhydrazone-based COF) photocatalysis (Liu et al., 2017). In addition, an imine-based COF containing triazine units, **COF-JLU5**, was used for the same CDC reaction, allowing a larger scope of THIQs and nucleophiles (phosphite and malonates) (Zhi et al., 2017). Also, two bidimensional imine-based COFs containing the photoactive units triphenylamine and tetraphenylethylene were employed for CDC reaction (Kang et al., 2020).

## Photocatalytic Oxygen Evolution Reaction

Formation of oxygen molecule from oxidation of water is a very challenging goal due to a large thermodynamic penalty and the complexity of reaction kinetics involved in the four-electron oxidation process. Generally, the commonly reported mechanism for the OER process implies oxidation of M-OH_2_ starting precursors forming a high-valent Metal oxo or Metal-hydroxo species, depending on the nature of metal center. Then O-O bond formation results from a bimetallic MO(H)···(H)OM association, or from monometallic MO(H)···OH_2_ reactivity. Further oxidation of peroxo species results of O_2_ generation. Overall, two consecutive bielectronic oxidative steeps should occur simultaneously to O-O bond formation. Therefore, redox-active metal centers are generally required to mediate on these multistep pathways (Mandal et al., 2017).

**FIGURE 2:**
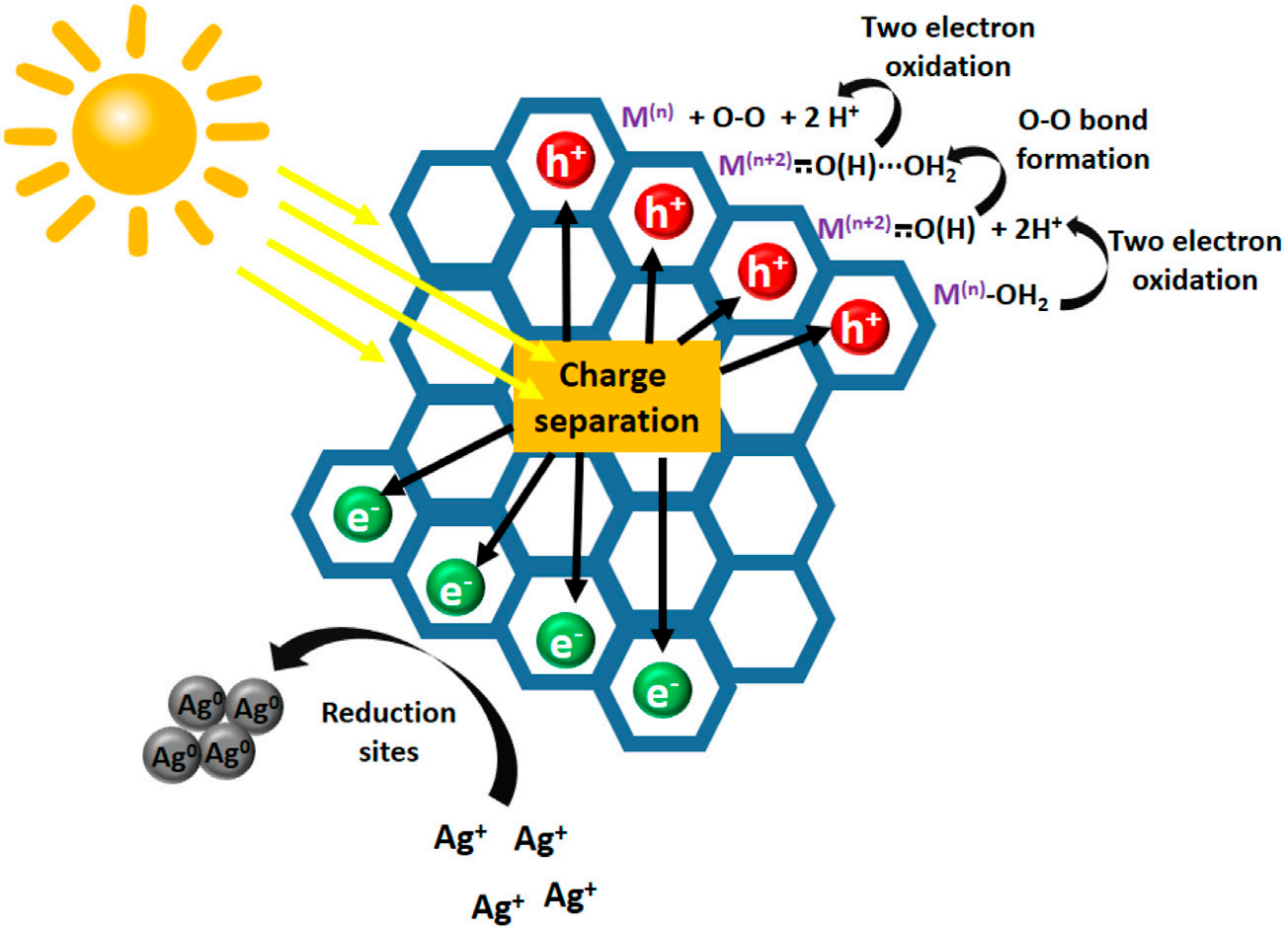
Schematic representation of pathway commonly claimed for photocatalytic oxygen evolution reaction.

Some examples of Covalent Organic Frameworks decorated with Ni (II), Fe(II), (Feng et al., 2020), Co(II) (Aiyappa et al., 2016; Jia et al., 2015), Ni_3_N (Nandi et al., 2016) or Nickel, Iron or Cobalt oxide nanoparticles (Mullangi et al., 2016) have been reported to act as OER electrocatalysts. Taking a step further, owing the tunable electronic properties of semiconductor COFs and CTFs, photocatalytic OER has been achieved as the result of synergy between photoactive material and catalytic metallic sites. The role of organic material generally consists on harvesting light, which enhances the redox reactivity of catalytic sites because of the efficient charge separation from light absorption. Therefore, water oxidation is possible using mild oxidizing agents, such as Ag^+^, which is typically used as final oxidizing reagent, only when light irradiation occurs (see Figure 2). Some examples of this kind of designs have been recently reported.

An outstanding example is a imine-based bipyridine containing COF that coordinates Co(II) centers. The photogenerated charge separation is produced by light absorption of the COF. The photogenerated electrons are captured by the Ag^+^ to produce Ag NPs, while the photogenerated holes result in generation of high valent cobalt species, which ultimately oxidizes the adsorbed H_2_O molecule to evolve oxygen (Chen et al., 2020).

Photocatalytic transformations require materials design that should allow to optimize the combination of light harvesting, exciton migration, electron transfer, charge separation and charge transport. According to a recent report, (Jin et al., 2019), this balance is better achieved by laminar fully π conjugated sp^2^ carbon-extended covalent organic frameworks, such as those based on cyano vinyldene groups, which can be used dispersed in water containing AgNO_3_ as final reducing agent and Co(NO_3_)_2_ as co-catalyst. Following a similar strategy, laminar COFs were obtained through condensation of arylmethyl carbon atoms in 3,5-dicyano-2,4,6-trimethylpyridine and linear/trigonal aldehyde monomers, generating *trans*-disubstituted C=C linkages. The corresponding Csp^2^ jointed pyridinyl crystalline honeycomb-like frameworks are structurally related to graphitic carbon nitride (g-C_3_N_4_), which also has been reported as a photocatalysts (Wen et al., 2017). Interestingly, when loaded with Co(NO_3_)_2_, these materials exhibited visible-light activity for OER with oxygen evolution rates comparable to that of bulk g-C_3_N_4_ (Bi et al., 2019).

CTFs are materials closely related to C_3_N_4_, which have been applied for photocatalytic water splitting recently because of their semiconductive properties and their adjustable band gap, that can even be modulated by the method of choice for material’s synthesis (Kong et al., 2019). Irreversible nature of CTF formation is responsible for the amorphous nature of materials obtained. However, in many cases, the lack of crystallinity is not a significant drawback for the photocatalytic activity for OER. Simple CTFs with different chain lengths, containing variable quantity of benzyl units in the backbone were reported as good photocatalysts for OER when loaded with Co(NO_3_)_2_ (Lan et al., 2018) or with ruthenium oxide (Xie et al., 2018; Bi et al., 2015). Intriguingly, while the common understanding of OER is usually associated to the role of metal centers as mediators in electron transfer steeps coupled to O-O bond formation, oxygen evolution has been reported using CTF-0 material without any co-catalyst in the presence of Ag^+^ as the electron scavenger (Bi et al., 2015; Wen et al., 2017; Lan et al., 2018; Xie et al., 2018; Bi et al., 2019; Jin et al., 2019; Kong et al., 2019; Chen et al., 2020). Further mechanistic insights of this surprising process are expected.

## Future Perspectives and Final Remarks

Photoactivation of oxygen molecule mediated by Covalent Organic Frameworks, to achieve oxidation of organic substrates, is a field of research that achieved some degree of maturity, being reported the most common photocatalytic transformations. However, more complex systems, such as synthesis of asymmetric products, or cooperative multicomponent catalysis, still remain unexplored. Future developments on the photocatalytic use of COFs and Conjugated Organic Materials will require a fine tuning of molecular architectures with semiconducting properties. Control on linkages, building blocks, and end groups can serve to predesign materials able to facilitate charge separation and charge-carrier transport and preventing the charge recombination of photoexcited states.

The reticular chemistry scientific community has a clear bias toward the detailed structural elucidation and physical characterization. Thus, materials with poor crystallinities and scarce porosities are often discarded despite the photocatalytic functions that can be observed for amorphous materials. It is commonly assumed that ordered channels in crystalline materials with high surface areas facilitate mass transport. However, microporosity do not assure accessibility of reactants because diffusion into the internal regions of bulk porous materials is more difficult when the size particle is increased. In fact, when activity is related to single catalytic sites, results are improved by reducing the particle size, even if it implies a decrease on crystallinity and measured porosity (Zou et al., 2012; Aiyappa et al., 2016; Liu et al., 2017; Zhi et al., 2017; Wei et al., 2018; Jiménez-Almarza et al., 2019; Luis-Barrerra et al., 2019; Liu et al., 2020b; Feng et al., 2020; Kang et al., 2020; Shi et al., 2020). These observations indicate that catalytic processes generally occur in the pores that are closer to the particle surface. To this respect, it is significant that CTFs, which commonly show poor crystallinities and low surface areas, have been reported for several photocatalytic oxidations. Only when photoactivity is related with extended conjugation, and it is compromised by structural disorder, crystallinity has a positive impact on catalytic results (Jia et al., 2015; Mullangi et al., 2016; Nandi et al., 2016; Huang et al., 2017). Therefore, although structural integrity is interesting for some applications of COFs, research on photocatalytic COFs should concentrate on the main parameters related on the activity, which are not necessarily crystallinity and porosity.

The two main mechanistic possibilities of aerobic photooxidation of organic molecules are relatively well stablished via single oxygen or superoxide radical anion. The same material can usually perform through both mechanisms, although, one is generally the predominant for each specific material. Thus, when mechanistic studies are performed, the question that should be addressed is about what is the major process rather than just collect evidences of intermediates, which could be minor. Consequently, spectroscopic detection (for instance, through EPR) of transient species should be complemented with other experiments that can help to determine the specific contribution (electron transfer vs. energy transfer) of such intermediate to the overall catalytic outcome. A feasible way to address this issue is the use of specific scavengers that can selectively inhibit one or another pathway.

Uses of COFs in photocatalytic water oxidation have started to be explored very recently. However, owing the complexity of this process, little is known on the pathways followed for photocatalytic OER mediated by organic materials. The majority of the cases reported in literature consist on metal centered OER, while the role of the organic material is light harvesting. However, activities found for a metal-free system suggest that mechanistic landscape could be widened in future developments.

Overall, the state of the art shown in this overview confirms that photocatalytic applications of COFs for oxidation processes has a great growing potential. Modern chemistry challenges such as artificial photosynthesis, energy conversion, design of environmentally benign processes or the production of high added value products, should be further addressed by means of the design of new photocatalytic organic materials.
